# Neuromorphic Event-Based Generalized Time-Based Stereovision

**DOI:** 10.3389/fnins.2018.00442

**Published:** 2018-07-02

**Authors:** Sio-Hoi Ieng, Joao Carneiro, Marc Osswald, Ryad Benosman

**Affiliations:** ^1^Institut National de la Santé et de La Recherche Médicale UMRI S 968, Sorbonne Universités, UPMC Universités Paris, UMR S 968, Centre National de la Recherche Scientifique, UMR 7210, Institut de la Vision, Paris, France; ^2^Institute of Neuroinformatics, University and ETH Zurich, Zurich, Switzerland

**Keywords:** asynchronous acquisition, time-pulse encoding, event-based stereovision, frameless vision, asynchronous vision

## Abstract

3D reconstruction from multiple viewpoints is an important problem in machine vision that allows recovering tridimensional structures from multiple two-dimensional views of a given scene. Reconstructions from multiple views are conventionally achieved through a process of pixel luminance-based matching between different views. Unlike conventional machine vision methods that solve matching ambiguities by operating only on spatial constraints and luminance, this paper introduces a fully time-based solution to stereovision using the high temporal resolution of neuromorphic asynchronous event-based cameras. These cameras output dynamic visual information in the form of what is known as “change events” that encode the time, the location and the sign of the luminance changes. A more advanced event-based camera, the Asynchronous Time-based Image Sensor (ATIS), in addition of change events, encodes absolute luminance as time differences. The stereovision problem can then be formulated solely in the time domain as a problem of events coincidences detection problem. This work is improving existing event-based stereovision techniques by adding luminance information that increases the matching reliability. It also introduces a formulation that does not require to build local frames (though it is still possible) from the luminances which can be costly to implement. Finally, this work also introduces a methodology for time based stereovision in the context of binocular and trinocular configurations using time based event matching criterion combining for the first time all together: space, time, luminance, and motion.

## 1. Introduction

Since the seminal work of Marr and Poggio (1977) and Julesz (1963) and other pioneers, stereovision has increasingly been studied. 3D perception has become one of the key technologies for several tasks, such as autonomous driving, grasping, gaming, drone navigation,etc. However, there are still very few vision-based applications in uncontrolled light conditions and unstructured environements. There are currently several available sensors providing 3D perception such as laser range finders or laser scanners, time-of- flight (TOF) cameras, ultrasonic detectors, radar, light-section, and structured light as well as passive technologies, including structure from motion, optical flow, and stereo vision. Current state-of-the-art methods for autonomous driving (Levinson and Thrun, 2010; Markoff, 2010) use reflectivity measurements from 3D LIDAR scanners to create an orthographic map of ground-plane reflectivities. LIDAR are becoming the gold standard for outdoor navigation. They are however slow, computationaly more demanding and far more power hungry than cameras. The cost of 3D LIDAR scanners is also prohibitive for consumer market automobiles. Cameras provide a low-cost means to generate extremely rich, dense data that is suitable for generating dense 3D maps, however the low dynamic range and current low frame-rates of conventional cameras are still a major bottleneck for their use. Conventional camera-based stereovision is also considered unreliable specially when dealing with uncontrolled light conditions. This is mainly due to the mode of operation of state-of-the-art image sensors that is useful and efficient for exactly one thing: photography, i.e., for taking an image of a still scene. There is a widespread and ingrained belief that acquiring series of images at some rate is a good way to capture visual motion. This conviction is seemingly supported by the way movies are made for human observers. The observation that visual motion appears smooth and continuous if viewed above a certain frame-rate is, however, more related to characteristics of the human eye and visual system than to the quality of the acquisition and encoding of the visual information as a serie of images (Akolkar et al., 2015). As soon as changes or motions are involved, which is the case for most machine vision applications, the universally accepted paradigm of visual frame acquisition becomes fundamentally flawed. If a camera observes a dynamic scene, no matter where the frame-rate is set to, it will always be wrong. Because there is no relation whatsoever between dynamics present in a scene and the chosen frame-rate, over-sampling and/or under-sampling occur, and moreover both usually happen at the same time. When acquiring a natural scene with a fast moving object, e.g., a ball thrown in front of a static background with a standard video camera, motion blur and displacement of the moving object between adjacent frames will result from under-sampling the object, while repeatedly sampling and acquiring static background over and over again will lead to large amounts of redundant, previously known data. As a result, the scene is under- and over-sampled at the same time! Interestingly, this far-from-optimal strategy of acquiring dynamic visual information has been accepted by the machine vision community for decades, likely due to the lack of convincing alternatives.

Recently, research in the field of Neuromorphic Engineering has resulted in a new type of vision sensors that acquire visual information in a very different way. These sensors are based on pixels that can optimize their own sampling depending on the visual information they individually receive. If scenes change quickly, the pixel samples at a high rate; if nothing changes, the pixel stops acquiring redundant data and goes idle until the scene changes significantly again in the sensors' field of view. These sensors introduce another paradigm of visual information acquisition: the pixels, instead of being driven by a fixed frequency that makes them work synchronously as in a classic frame-based sensor, are independent both in the samples acquisition times and the exposure durations. The data acquired that way is globally a time-continuous stream of visual information. In order to do so, each pixel defines the timing of its own sampling points in response to its visual input by reacting to changes of the amount of incident light. As a consequence, the sampling process is no longer governed by a fixed external signal defined in the time domain but by the signal to be sampled itself, or more precisely by the variations of the signal in the amplitude domain. Mahowald (1992) introduced the early form of the neuromorphic vision sensor that lead to several variations of what are presently known as the event-based vision sensors: (Lichtsteiner et al., 2006; Serrano-Gotarredona and Linares-Barranco, 2013) are encoding temporal contrasts asynchronously in the form of pulses called events. Newer generations of event-based sensors have either integrated a synchronous frame mode (Berner et al., 2013) or have implemented a level crossing sampling mechanism to captur and encode luminance in an asynchronous way (Posch et al., 2011).

The Asynchronous Time-based Image Sensor (Posch et al., 2011) used in this paper is an asynchronous camera that contains an array of independently operating pixels that combine an asynchronous level-crossing detector and a separate exposure measurement circuit. Each exposure measurement by an individual pixel is triggered by a level-crossing event. Hence each pixel independently samples its illuminance upon detection of a change of a certain magnitude in this same luminance, thus establishing its instantaneous gray level after it has changed. The result of the exposure measurement (i.e., the new gray level) is asynchronously output off the sensor together with the pixel‘s coordinates in the sensor array. As a result, image information is not acquired frame-wise but continuously, and conditionally, only from parts of the scene where there is new visual information. Or in other words, only information that is relevant–because it has changed–is acquired, transmitted, stored and eventually processed by machine vision algorithms. Pixel acquisition and readout times of microseconds to milliseconds are achieved, resulting in temporal resolutions equivalent to conventional sensors running at tens to hundreds of thousands frames per second. The implications of this approach for machine vision can hardly be overstated. Now, for the first time, the strict temporal resolution vs. data rate tradeoff that limits all frame-based vision acquisition can be overcome. Visual data acquisition simultaneously becomes fast and sparse. Obviously the advantages of acquiring dynamic vision data this way, i.e., ultra-high-speed operation combined with reduced power consumption, transmission bandwidth and memory requirements, do not end at the acquisition stage. All subsequent processing strongly benefits from the fact that the sensors encode visual dynamics into highly resolved spatio-temporal patterns of “events,” representing the relevant features of motion such as moving object contours and trajectories virtually in continuous time.

The event-based formulation of stereovision has already produced striking results in stereovision. The use of time allowed the reformulation of the epipolar constraint as a time coincidence phenomenon as shown in Benosman et al. (2011). Epipolar lines defining the relation established by two vision sensors appear as stuctures of co- occurent events. This methodology can be naturally extended to solve the problem of 3D matching and reconstructions from events as introduced in Rogister et al. (2011) and Carneiro et al. (2013). Event-based stereovision techniques based on changes events assume no luminance in the encoding of the visual information since the camera used in these works only provides the time and the sign of detected changes. This last consideration is the main motivation splitting this work from many others that can be found in literature in event-based stereo vision as Schraml et al. (2007), Kogler et al. (2009), Schraml et al. (2010), Dominguez-Morales et al. (2011), and Belbachir et al. (2012). These works apply matching methodes based on events accumulation to build frames so standard binocular vision techniques can be applied. We are intentionally getting away from these approaches, as building frames induces usually a lost in temporal precision and is not allowing us to exploit the event-based representation at its fullest potential. In Kogler et al. (2011), the authors also claimed the benefit of using accurate time information provided by the sensor instead of simply accumulating events to build local/global frames. They developed an event matching algorithm that is pretty similar to the one in Rogister et al. (2011). At the time, the community started to focus more on how to infer depth from the temporal information rather than just the spatial information obtained via events accumulation. Piatkowska et al. (2013) implemented an event-based form of a “cooperative” computation of depth coupled with a winner-take-all mechanism to match events temporaly close and spatially constrained by the epipolar geometry satisfied by the event-based vision sensors. This cooperative technique has been actually initiated by the early work of Marr and Poggio (1976) on frame-based cameras.

This paper completes and generalizes previous work on event-based stereovision by introducing a new approach to solve pure event driven stereo matcting. It combines for the first time: precise timing (Rogister et al., 2011), local motion consistency (Benosman et al., 2012) and light consistency in the temporal domain (Posch et al., 2011). As we will show, the extremly high temporal resolution (up to *n*s) of the acquisition and encoding process allows the formulation of stereovision as a coincidence detection problem in the temporal domain, in which time also encodes for luminance.

## 2. Time encoded imaging

**Figure 1:**
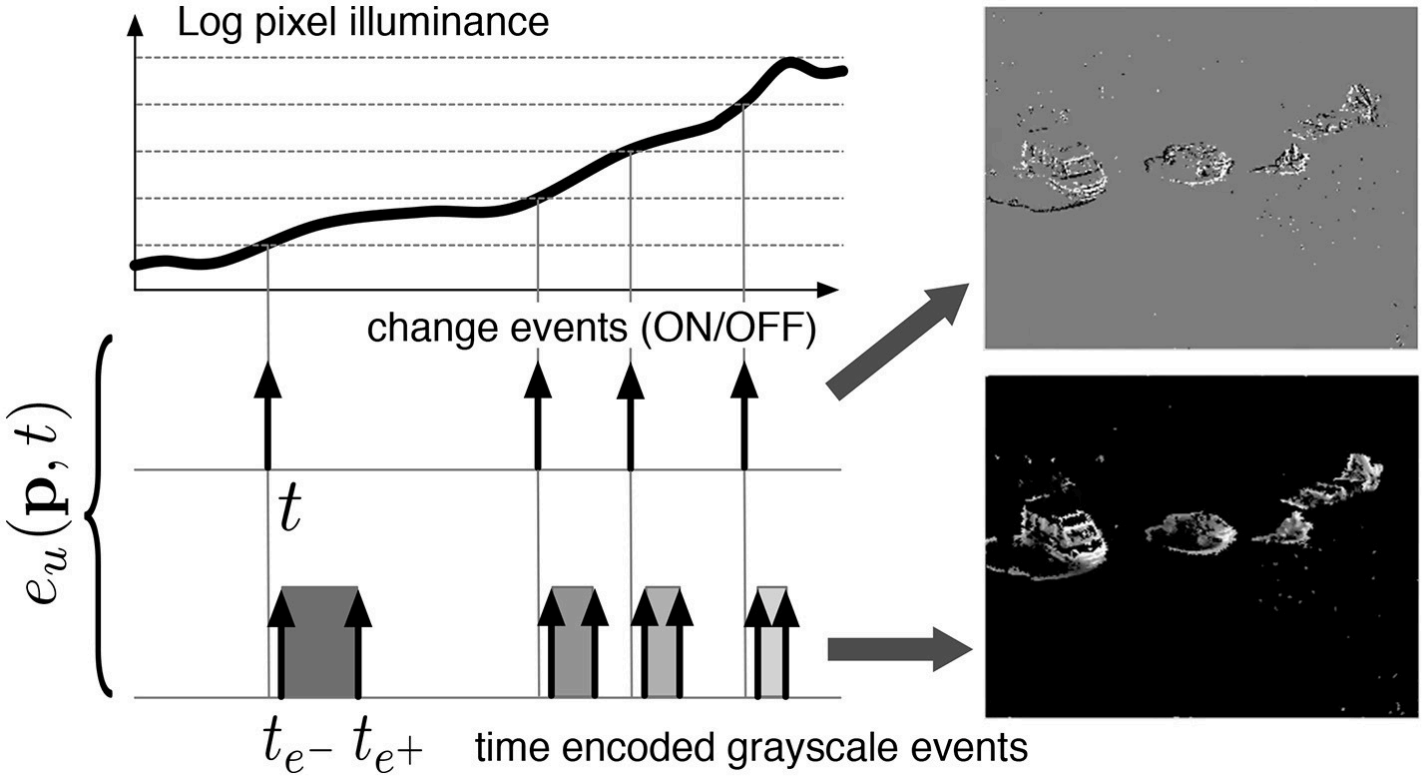
Functional diagram of an ATIS pixel (Posch et al., 2011). Two types of asynchronous events, encoding change and brightness information, are generated and transmitted individually by each pixel in the imaging array.

The Asynchronous Time-based Image Sensor (ATIS) used in this work is a time-domain encoding image sensor with QVGA resolution (Posch et al., 2008, 2011). The sensor contains an array of autonomous pixels that combine an illuminance change detector circuit and a conditional exposure measurement block. As shown in the functional diagram of an ATIS pixel in Figure 1, the change detector individually and asynchronously initiates the measurement of an exposure/gray level value only if—and immediately after—a brightness change of a certain magnitude has been detected in the field-of-view of the pixel at time *t*. ATIS encodes visual information as a stream of events where each event *e*_*u*_(**p**, *t*) output by camera *u* is defined by its image coordinates **p** = (*x, y*)^*T*^, time of change *t*, polarity *pol* and luminance information encoded between two times that define the begining and end of the light integration written respectively $t_{e^{-}}$ and $t_{e^{+}}$. The exposure measurement circuit in each pixel individually encodes the absolute instantaneous pixel illuminance into the timing of asynchronous event pulses, more precisely into inter-event intervals.

An event *e*_*u*_(**p**, *t*) can then be defined as quadruple:

(1) $$e_{u}(p,t)=(t,p,I_{u},pol)\text{ with }\{\begin{array}{l} t\\ p={\left(x,y\right)}^{T}\in R_{u}\\ I_{u}(p,t)=\frac{1}{t_{e^{+}}\;-\;t_{e^{-}}}\\ pol=sign\left(\frac{\partial I_{u}(p,t)}{\partial t}\right) \end{array},$$

**Figure 2:**
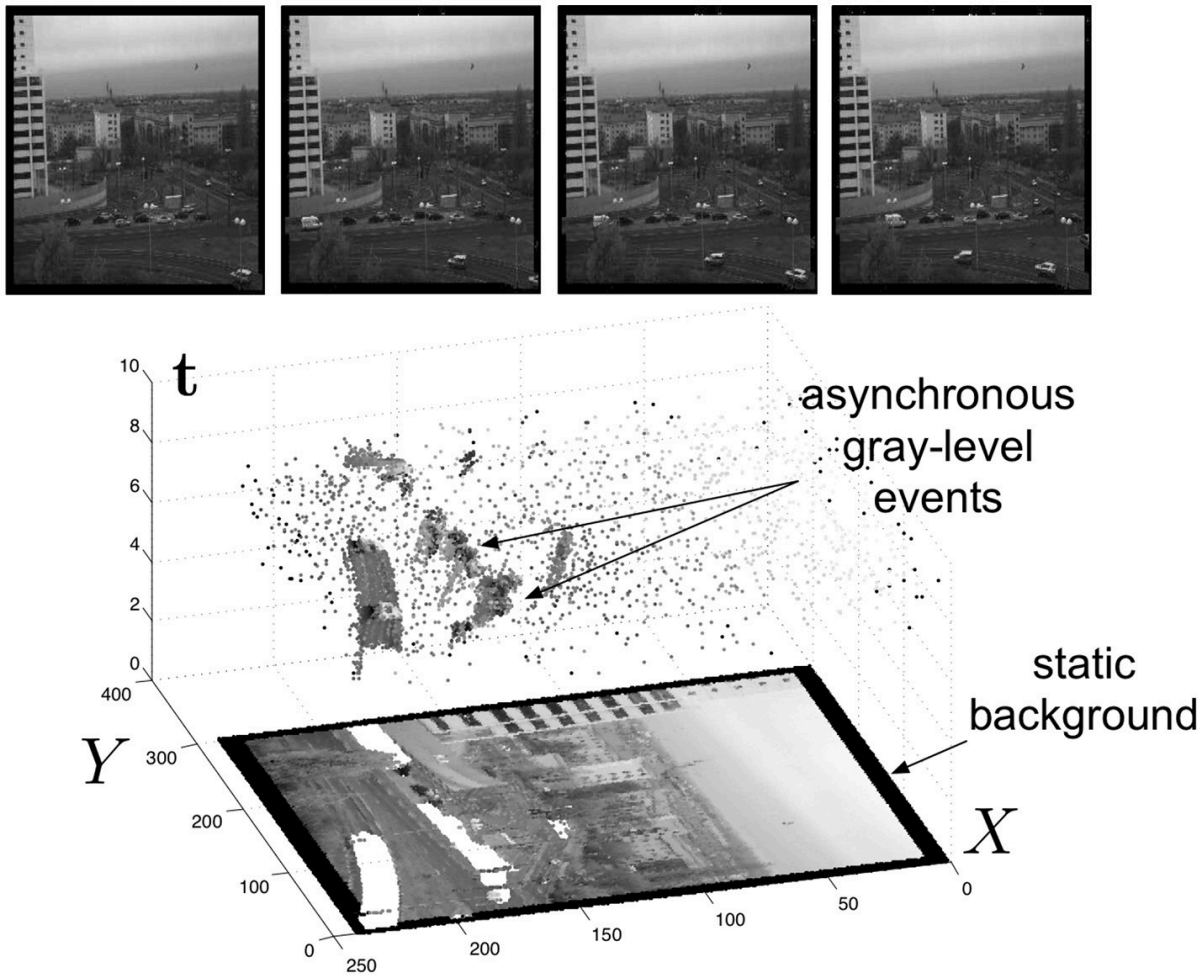
(Lower part) The spatio-temporal space of imaging events: Static objects and scene background are acquired first. Then, dynamic objects trigger pixel-individual, asynchronous gray level events after each change. Frames are absent from this acquisition process. Samples of generated images from the presented spatio-temporal space are shown in the upper part of the figure.

*I*_*u*_(**p**, *t*) is the luminance that can be computed directly as the inverse of $t_{e^{+}}-t_{e^{-}}$, where $t_{e^{-}}$ and $t_{e^{+}}$ being respectively the starting and the finishing timestamps of the integration. Finally, $R_{u}$ designates the focal plane of camera *u*. Since the ATIS is not clocked like conventional cameras, the timing of events can be conveyed with a temporal resolution at the order of 1 ms. The time-domain encoding of the intensity information automatically optimizes the exposure time separately for each pixel instead of imposing a fixed integration time for the entire array, resulting in an exceptionally high dynamic range of 143 db and an improved signal to noise ratio of 56 db. The polarity *pol* is representing the direction of the induced luminance change from its previous value. Events can therefore assume a single value 1 or −1 if they represent respectively an increase or decrease of the luminance. Figure 2 shows the general principle of asynchronous imaging spaces. Frames are absent from this acquisition process. They can however be reconstructed, when needed, at frequencies limited only by the temporal resolution of the pixel circuits, up to hundreds of kiloframes per second if the scene is sufficiently bright. In low light condition, the luminance integration time is introducing additional latencies which might reduce frame-rate drastically. Figure 2 (top) shows samples of such generated gray level frames. Static objects and background information, if required, can be recorded as a snapshot at the start of an acquisition henceforward moving objects in the visual scene describe a spatio-temporal surface at very high temporal resolution (see Figure 2 bottom).

## 3. Materials and methods

### 3.1. Event-based stereo matching

A light intensity variation at a given 3D point **X** will be projected onto the image plane $R_{u}$ at the location **p** according to:

(2) $$\left(\begin{array}{c} p\\ 1 \end{array}\right)=P_{u}\left(\begin{array}{c} X\\ 1 \end{array}\right),$$

where *P*_*u*_ is the perspective projection matrix of camera *u*.

In what follows we will consider two stereo cases: binocular and trinocular. Considering a trinocular configuration usually opens the path for a higher number of cameras as shown in Carneiro et al. (2013). We have shown that stereo vision can be computed solely from a purely temporal matching of events constrained by epipolar geometry both for binocular (Rogister et al., 2011) and trinocular stereo configurations (Carneiro et al., 2013). The principle being that pixels sending events at the same time are potentially observing the same moving stimulus. Results show however that higher amount of correct matches can be reached if more constraints are added on the matching (Camuñas-Mesa et al., 2014). Matching raw events is always prune to errors as raw events only carry temporal information. We will generalize the initial work by providing a more general scheme going beyond the combined use of time and epipolar geometry. We will introduce more criterions such as luminance, motion all computed from time and more importantly, we will show how luminance can be used to derive a time coincidence detection that allows to increase the matching performances when combined with motion information derived from teh events.

### 3.2. Spatial criterion

Let us consider *F*_*uv*_ as the fundamental matrix that maps events between cameras *u* and *v*, **l**_*uv*_(**p**) for $\text{p}\in R_{u}$, is the epipolar line on the image plane $R_{v}$ defined as:

(3) $$\begin{array}{l} R_{u}\to \;{\mathbb{P}}^{2}\\ p\mapsto l_{uv}=F_{uv}\left(\begin{array}{c} p\\ 1 \end{array}\right), \end{array}$$

where ℙ^2^ is the projective space of ℝ^2^. Equation 3 is the mathematical form of the epipolar constraint. It means that for a pair of vision sensors defining the fundamental matrix *F*_*uv*_, any pixel in camera *u* is mapped to a line **l**_*uv*_ in $R_{v}$.

The 3-tuple of matching events produced by three sensors *u, v*, and *w*, is defined as *m* = {*e*_*u*_(**p**, *t*_*u*_), *e*_*v*_(**q**, *t*_*v*_), *e*_*w*_(**r**, *t*_*w*_)} of events generated at pixels **p**, **q**, and **r**, on the intersection of the epipolar lines in respectively image planes $R_{u}$, $R_{v}$ and $R_{w}$. If the intersections of epipolar lines are exact, a formal definition of a 3-tuple is:

(4) $$\hat{m}=\left\{e_{i}(\hat{x},t_{i})|\hat{x}=l_{ji}\cap l_{ki}\right\},$$

where for an event *e*_*u*_, {*i, j, k*} is any circular permutation of {*u, v, w*} and $\hat{\text{x}}$ is the exact intersection of two epipolar lines. $\hat{\text{x}}$ is, up to some estimation error, close to the actual pixel **x** captured by the camera *i* (i.e., **x** ∈ {**p**, **q**, **r**}). The geometrical error for a given match is defined as the mean distance between the intersection of epipolar lines and the matched point at each retina and it reflects how well a match respects the epipolar constraints:

(5) $$E_{G}(m)=\frac{1}{3\epsilon_{g}}\left(\left|p-\hat{p}|+|q-\hat{q}|+|r-\hat{r}\right|\right).$$

ϵ_*g*_ is a normalizing scalar which represents the maximum allowed geometric distance. This maximum allowed distance defines which events are considered as potential candidates and therefore if $|\text{x}-\hat{\text{x}}|>\epsilon_{g}$, the match is discarded automatically.

For binocular matching, there is no epipolar line intersection, instead the geometrical error is given by the distance from candidate points to epipolar lines:

(6) $$E_{G}(m)=\frac{d(p,l_{uv})+d(q,l_{vu})}{2\epsilon_{g}}$$

*d*(**x**, **l**) is the distance from point **x** to the epipolar line **l**.

### 3.3. Temporal criterion

On the time domain, the matching is achieved by identifying events which occur at the same time on all sensors. If a given stimulus **X** is changing luminance that is detected at *t* by sensors *u*, *v* and *w*, events *e*_*u*_(**p**, *t*_*u*_), *e*_*v*_(**q**, *t*_*v*_), and *e*_*w*_(**r**, *t*_*w*_) are then generated, where *t* ≈ *t*_*u*_ ≈ *t*_*v*_ ≈ *t*_*w*_, because of different retina latencies. However, we can define matching events as the ones generated at the closest temporal distance by minimizing the temporal matching error

(7) $$E_{T}(m)=\frac{\left|t_{u}-t_{v}|+|t_{u}-t_{w}\right|}{2\epsilon_{t}}$$

where ϵ_*t*_ is a normalizing scalar which represents the maximum temporal distance error. Similarly, in the binocular case we have:

(8) $$E_{T}(m)=\frac{\left|t_{u}-t_{v}\right|}{\epsilon_{t}}$$

### 3.4. Generalized time criterion

Let events *e*_*u*_(**p**, *t*_*u*_), *e*_*v*_(**q**, *t*_*v*_) be events generated from a moving 3D point. A event *e*_*u*_ generates, as shown in Figure 1, three events, *t*_*u*_, the change event and $t_{e^{-},u},t_{e^{+},u}$ the luminance integration events. Let $I_{u}=\left\{t_{u},t_{e^{-},u},t_{e^{+},u}\right\}$ be the set of event trains related to *e*_*u*_, these can be represented mathematically as:

(9) $$l_{u}(t)=\sum_{i\;=\;1}^{n}\delta (t-I_{u}(i)),$$

where *i* indexes one of the three events $t_{u},t_{e^{-},u}$ or $t_{e^{+},u}$.

**Figure 3:**
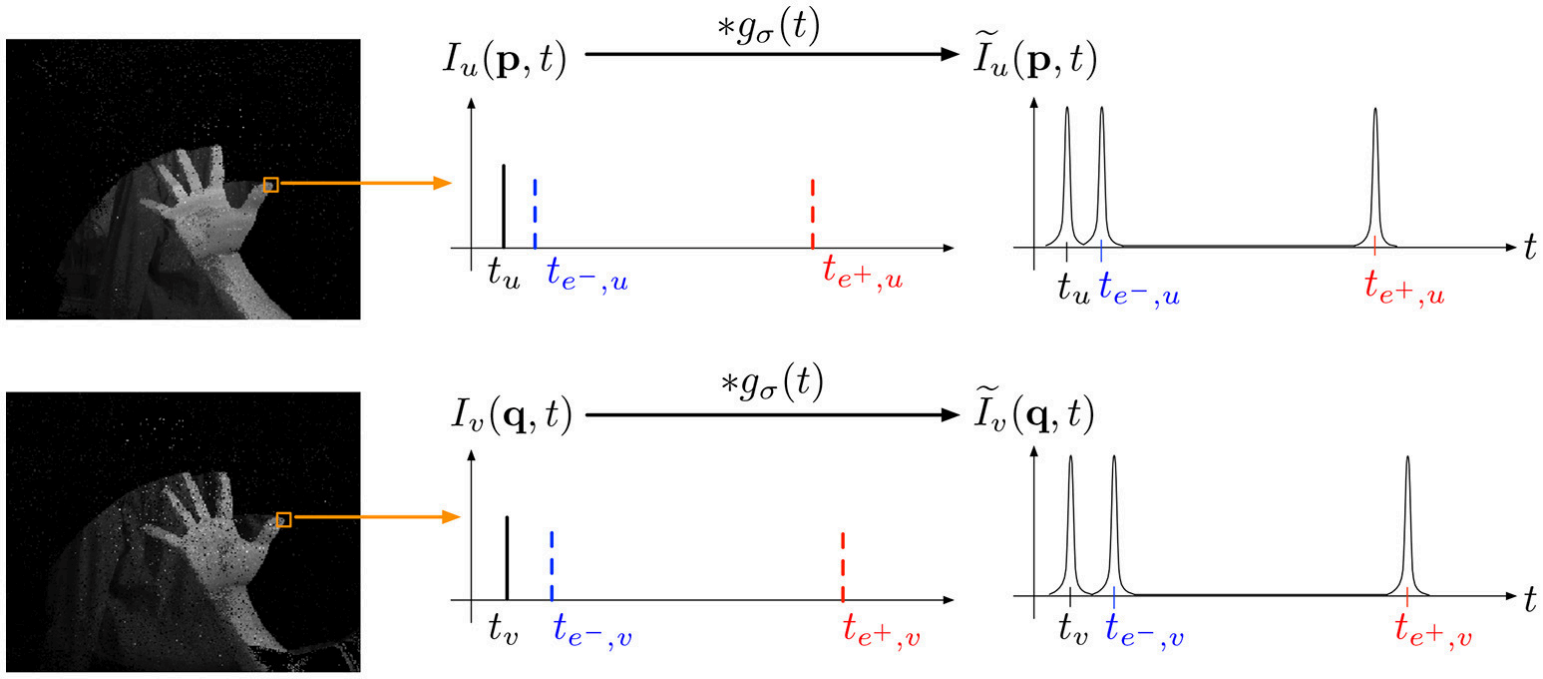
As intensities are time-coded by sets of three events (a change event, an integration start and an end integration event), finding the best match of a given intensity is equivalent to identifying coincidences of the three events timestamps. In term of spike train, we are maximizing the similarity measure defined in Schreiber et al. (2003) by convolving each spike train with a gaussian *g*(σ) of variance σ.

We can then define $\tilde{I}$ as the continuous function obtained by convolving *l* with a gaussian *g*(σ) of variance σ^2^ (see Figure 3). In practice, σ is set to have the three events represented by three non overlapping gaussians. Experiments show that for a variety of scenes $\sigma =\min\frac{\left(t_{e^{-}}-t,t_{e^{+}}-t_{e^{-}}\right)}{20}$ is a good choice. The similarity measure between two event trains ${\tilde{I}}_{u}\left(t\right)$ and ${\tilde{I}}_{v}\left(t\right)$ is then given by:

(10) $$E_{I}(m)=\frac{\int_{\omega }{\tilde{I}}_{u}(t){\tilde{I}}_{v}(t)dt}{\sqrt{\int_{\omega }{\tilde{I}}_{u}{\left(t\right)}^{2}dt}\sqrt{\int_{\omega }{\tilde{I}}_{v}{\left(t\right)}^{2}dt}},$$

where ω is the support of the convolved functions $\tilde{I}$ over some neighborhood ν.

As luminance is encoded in time, this criterion merges both change events and luminance in a single comparison. Luminance correlation is here expressed in time as two coincidences thus increasing the number of necessary coincidences between two incoming events by a factor of 3 compared to direct matching on change events as introduced in Rogister et al. (2011). This criterion can be extended and applied to a whole neighborhood around an incoming event. Considering a *n* × *n* neighborhood ρ, this increases the amount of coincidences by 3*n*^2^ thus providing more robust matchings. In practice, we are computing the sum of the *n*^2^ similarity measures as defined in (10), one for each pixel within the neighborhood:

(11) $$S(m)=\sum_{m_{i}\in \rho }E_{I}(m_{i}).$$

Two events are matched together among a list of possible candidates if the sum is maximal. The unified representation of time and luminance is also computationally efficient, the conventional convolution to compare luminance neighborhoods necessitate more computations that just detecting 3 coincidences. Considering the high dynamic range of the ATIS we are expecting to spare substantial computation namely because in a purely time based neural implementation time coincidence can be directly used to detect both coactivation of events and luminance correlation without the need of storing large digits. The use of time is showing here to be in perfect adequation with the precise time computational architecture and neuromorphic processors.

### 3.5. Motion criterion: time surface matching

Considering an incoming event *e*(**p**, *t*), we can then define two maps Σ_*e*_ and S_*e*_ that associate respectively to each **p**, the time *t* and the polarity *pol*:

(12) $$\begin{array}{c} \begin{array}{c} \Sigma_{e}:{\mathbb{R}}^{2}\to \mathbb{R}\\ p\mapsto t \end{array},\begin{array}{c} {\text{S}}_{e}:{\mathbb{R}}^{2}\to \{-1,1\}\\ p\mapsto pol \end{array} \end{array}$$

**Figure 4:**
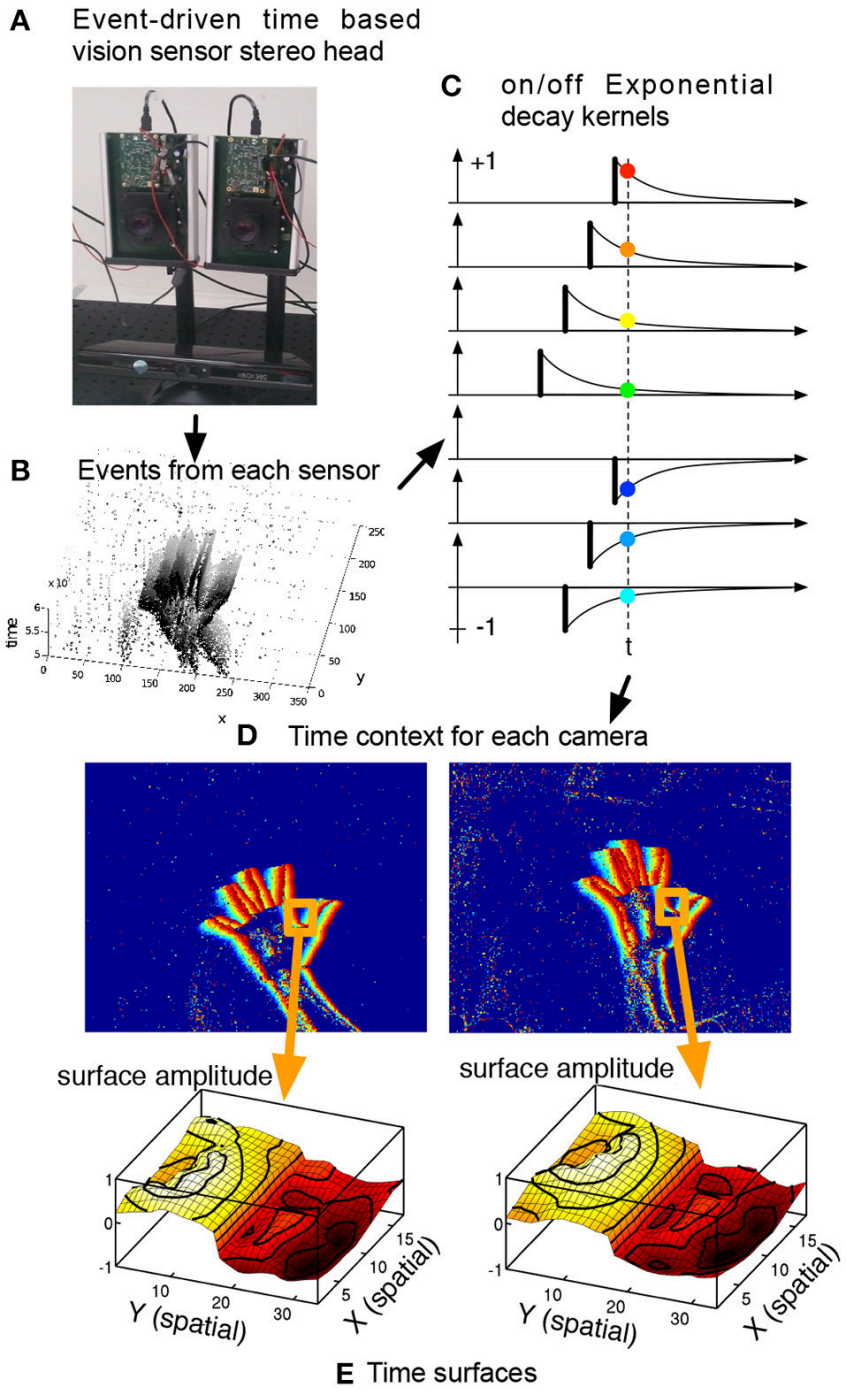
Definition of a time-surface from the spatio-temporal cloud of events. A time-surface describes the recent time history of events in the spatial neighborhood of an event. This figure shows how the time-surface is computed. The event-driven time-based vision sensor **(A)** is filming a scene and outputs events shown in **(B)**. Extracting a spatial neighborhood allows to build the event-context **(C)** associated with each incoming event. Exponential decay kernels are initiated at the time of arrival of events, their values at *t* constitute the time-surface itself. We consider the times of most recent events in the spatial neighborhood **(D)**. Extracting a spatial neighborhood allows to build the event-context introduced in **(C)** associated with that event. Exponential decay kernels are then applied to the obtained values, **(E)** shows these values as a surface.

We can now define the decaying time surface Γ_*e*_ at the current event time *t* and at any position **q**:

(13) $$\begin{array}{l} \Gamma_{e}:{\mathbb{R}}^{2}\times \mathbb{R}\to \mathbb{R}\\ \;(q,t)\mapsto {\text{S}}_{e}(q)\exp\left(\frac{\Sigma_{e}(q)-t}{\tau }\right), \end{array}$$

where τ is a time constant usually set experimentally between 5 and 20 ms. Time-surfaces provide a dynamic spatiotemporal context around an event related to motion which principle is shown in Figure 4. The exponential decay extends continuously the influence of past events (Figure 4C) and maintains the history of the activity in the neighborhood. The resulting surface is shown in Figure 4E. As one can see from the definition, |Γ_*e*_| is maximal and equal to 1 if **q** is actually where the last event occurs and converges to 0 the older the event that occured at **q**.

Let us define the spatio-temporal region around the spatio-temporal location of an event *e*_*u*_(**p**_*u*_, *t*) of size δ_*s*_ × δ_*s*_:

(14) $$\nu (e_{u})=\{\Gamma_{e}(p_{i},t)||p_{i}-p_{u}|\leq \delta_{s}\}.$$

If two events *e*_*u*_ and *e*_*v*_ are matched, their motion consistency can be computed by correlating their corresponding time surfaces. An energy cost function can be defined imposing penalties on motion disparity such that:

(15) $$E_{M}(m)=1-\frac{1}{2}\sum_{e_{u},e_{v}}\frac{\left(\nu (e_{u})-\bar{\nu (e_{u})})(\nu (e_{v})-\bar{\nu (e_{v})}\right)}{\left|(\nu (e_{u})-\bar{\nu (e_{u})}||(\nu (e_{v})-\bar{\nu (e_{v})}\right|}$$

where $\bar{\nu \left(e_{u}\right)}$ is the mean value of Γ_*e*_ over a neighborhood ν around an event *e*_*u*_.

## 4. Results

We presented four independent matching contraints:

- *E*_*G*_, spatial geometric consistency (Equation 5),
- *E*_*T*_, time consistency (Equation 7),
- *E*_*I*_, luminance consistency (Equation 10),
- *E*_*M*_, motion consistency (Equation 15).

We can define a temporal energy cost function written here as a summation of all temporal criterion:

(16) $$\tilde{E}(m)=E_{G}(m)+E_{T}(m)+E_{M}(m)+E_{I}(m),$$

such that,

(17) $$m(e_{u})=\underset{m_{i}\in M}{\text{argmin}}\;(\tilde{E}(m_{i})),$$

where M is the set of 3-tuple *m*_*i*_ within a spatiotemporal volume centered to the most recent events generated by any cameras *u, v* or *w*. Its size is defined by the maximum pixel error and the maximum temporal error tolerated.

The combination *E*_*G*_(*m*_*i*_) + *E*_*T*_(*m*_*i*_) is the initial method for event based matching introduced in Rogister et al. (2011). In order to study the effect of each additional criterion, we consider four *Ẽ* matching criterions:

- basic:
  (18) $$\tilde{E}(m_{i})=E_{G}(m_{i})+E_{T}(m_{i}),$$
- basic and motion:
  (19) $$\tilde{E}(m_{i})=E_{G}(m_{i})+E_{T}(m_{i})+E_{M}(m_{i}),$$
- basic and luminance:
  (20) $$\tilde{E}(m_{i})=E_{G}(m_{i})+E_{T}(m_{i})+E_{I}(m_{i}),$$
- basic, motion, and luminance
  (21) $$\tilde{E}(m_{i})=E_{G}(m_{i})+E_{T}(m_{i})+E_{M}(m_{i})+E_{I}(m_{i}).$$

In what follows we will ommit writing the basic form *E*_*G*_(*m*_*i*_) + *E*_*T*_(*m*_*i*_) in the notations except when it concerns directly the basic form itself, so that the reader can more easily focus on the contribution of every new criterion.

**Figure 5:**
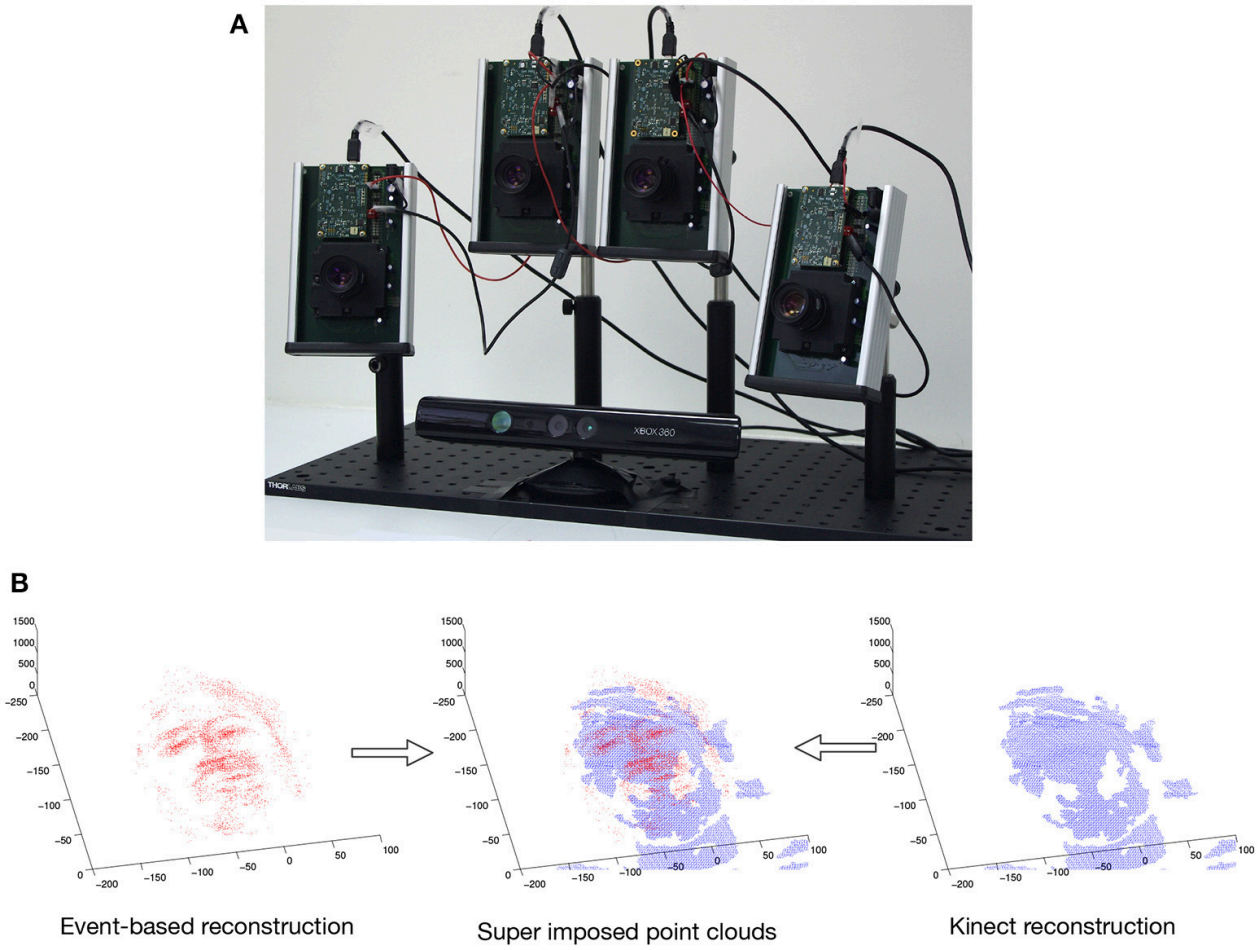
**(A)** Multicamera setup composed of 4 ATIS sensors and a Microsoft Kinect sensor for ground-truth. **(B)** Point cloud built from the events and point cloud from the Kinect super-imposed: a point from the event is paired to a point from the Kinect if their relative distance is the smallest.

The experimental setup shown in Figure 5A consists of a multi-camera rig of ATIS cameras and a Microsoft Kinect sensor. We consider binocular and trinocular configurations. The fourth camera is used as a backup and for future multi-camera studies. Cameras are synchronized. They are also calibrated using the toolbox introduced in Bouguet (2008). The Microsoft Kinect sensor is also calibrated with the multi-camera system, it provides the 3D ground-truth. We test the influence of these criterions independently and present 3D reconstruction results for temporal matching windows between 1 and 7 ms and matching pixel errors of 1–4 pixel. The matching time window interval compensates for the non idealities of the sensor, while the spatial pixel error reflects the distance to the epipolar line. In principle the calibration provides a subpixel spatial error, however in mobile applications, vibrations and collisions usually affect the calibration. The 3D reconstructions are computed and evaluated for each of the four criterion. In order to evaluate the accuracy of the results, each 3D point cloud reconstructed from the events is compared to the 3D point clouds produced by the Kinect within the same time slot (see Figure 5B). A point from the event-based point cloud is paired to the closest point from the Kinect. From this hypothesis, we define two measures to quantify the accuracy of the computed reconstructions:

- The reconstruction error is computed as the mean distance between reconstructed and closest ground truth points. Then the error is normalized by the maximum width of the object. This value tells how close the computed reconstruction is from the Kinect's point cloud.
- The number of wrong matches is given by the total number of points which distance to its corresponding closest point in the Kinect's point cloud is larger than 10%. This measure evaluates the amount of noise surrounding the recovered shape.

The dataset is made of a sequence showing a person in a standard but non blinking lighting condition, in a room providing uniform background. The event-based sensors are set to capture the head on their fields of view (fov) and the person is asked to move without specific constraint, within the fov of the sensors. The recording is 10 s long and is generating an average of 450 k events/s. One has to keep in mind that this events rate is not only dependent on the sensors setting but mainly scene dependent.

### 4.1. Binocular matching

**Figure 6:**
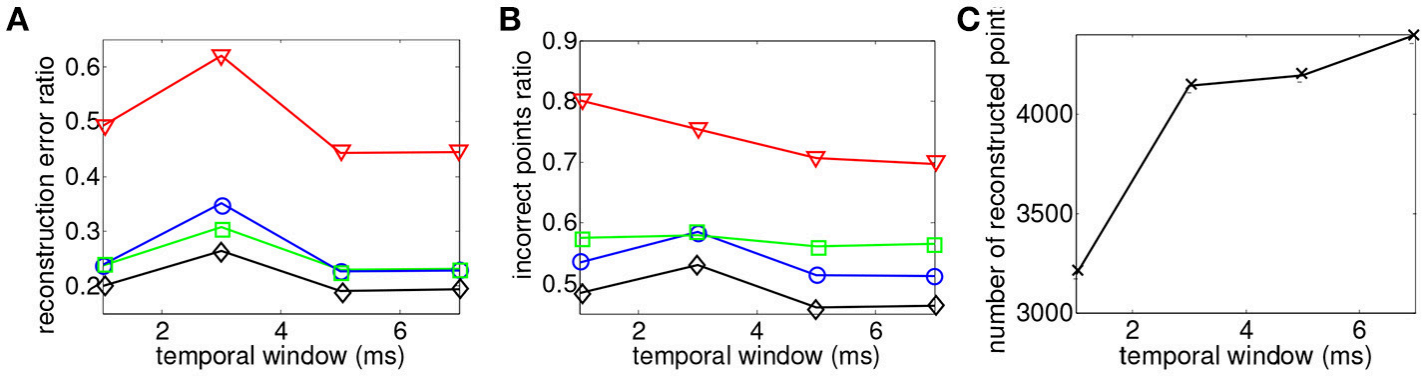
Comparison of reconstruction accuracy and errors with each matching cost function and variable matching time window applied to the asynchronous event-based binocular stereo matching (Rogister et al., 2011). Red line (triangle) represents geometrical and temporal minimization (*E*_*G*_ + *E*_*T*_), green line (square) represents the addition of motion (*E*_*G*_ + *E*_*T*_ + *E*_*M*_), blue line (circle) represents the addition of luminance (*E*_*G*_ + *E*_*T*_ + *E*_*I*_) and black (diamond) represents the addition of motion and luminance (*E*_*G*_ + *E*_*T*_ + *E*_*M*_ + *E*_*I*_). **(A)** Accuracy of 3D reconstruction **(B)** Amount of incorrect points **(C)** Total amount of reconstructed points with different matching time windows.

3D reconstruction is obtained from operating the asynchronous event-based binocular stereo matching algorithm over the sequence encoding a moving human face. The maximum matching geometrical distance is fixed such that ϵ_*g*_ = 1 pixel while the matching time-window parameter is tested for values ranging from 1 ms ≤ ϵ_*t*_ ≤ 7 ms at fixed intervals of 2 ms. 3D reconstructions are obtained with minimization of each of the four proposed energy cost functions. Results are presented in Figure 6, they show the influence of the matching time window width in the binocular configuration.

Figure 6A shows that the reconstruction accuracy remains almost constant regardless to the time window width for all criterion. The basic criterion *E*_*G*_ + *E*_*T*_ (shown by the red curve) achieves very poor results with reconstruction accuracy of 50%. Furthermore Figure 6B shows that 75% of recovered 3D points are wrong matches meaning that this method produces very noisy and inaccurate point clouds. The use of any of the other proposed cost functions shows far better results. The addition of *E*_*M*_ or *E*_*I*_ provides equivalent improvements in terms of accuracy with average reconstruction errors of around 25%. However, *E*_*M*_ seems to produce slightly more wrong matches than *E*_*I*_ with respectively 57% average amount of wrong matches against 53% given by the second function. The addition of both motion and flow *E*_*M*_ + *E*_*L*_ provides the best results with an average of 20% reconstruction error and <50% of wrong matches. The increase of the time-window does not seem to have an influence on the accuracy or the percentage of wrong matches as both remain constant. However, the number of reconstructed points improves when the time window is increased.

**Figure 7:**
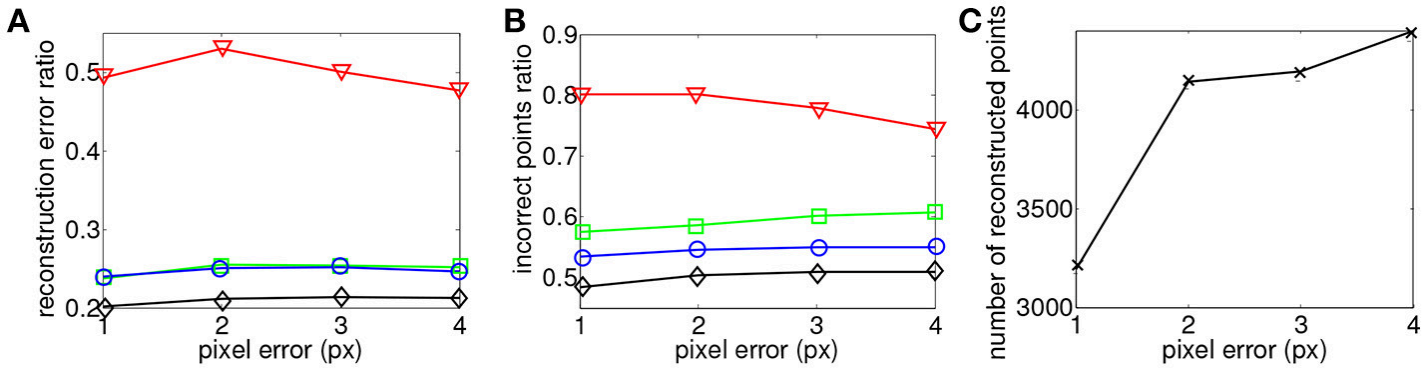
Comparison of reconstruction accuracy with each constraint and a range of distances to epipolar lines using the binocular configuration. Red line (triangle) represents *E*_*G*_ + *E*_*T*_, green line (square) represents the addition of motion (*E*_*M*_), blue line (circle) represents the addition of luminance (*E*_*I*_) and black (diamond) representsthe addition of motion and luminance (*E*_*M*_ + *E*_*I*_). **(A)** Accuracy of the 3D reconstruction **(B)** Amount of incorrect points **(C)** Total amount of reconstructed points.

Similar results are obtained when studying the distance to epipolar lines value. In this case, the maximum matching temporal distance is fixed such that ϵ_*t*_ = 1 ms while the matching pixel error parameter is tested for values ranging 1 pixel ≤ ϵ_*g*_ ≤ 4 pixel. Results are shown in Figure 7. The reconstruction accuracy and the amount of wrong matches remain constant. The amount of reconstructed points increases when the distance to the epipolar line is increased. The same conclusions on the effects of noise also apply to this case. Spatial and temporal matching distances do not seem to have an effect on the accuracy of the reconstruction. However, when these criterions are loosened the number of reconstructed points and amount of produced noise increases.

### 4.2. Trinocular matching

**Figure 8:**
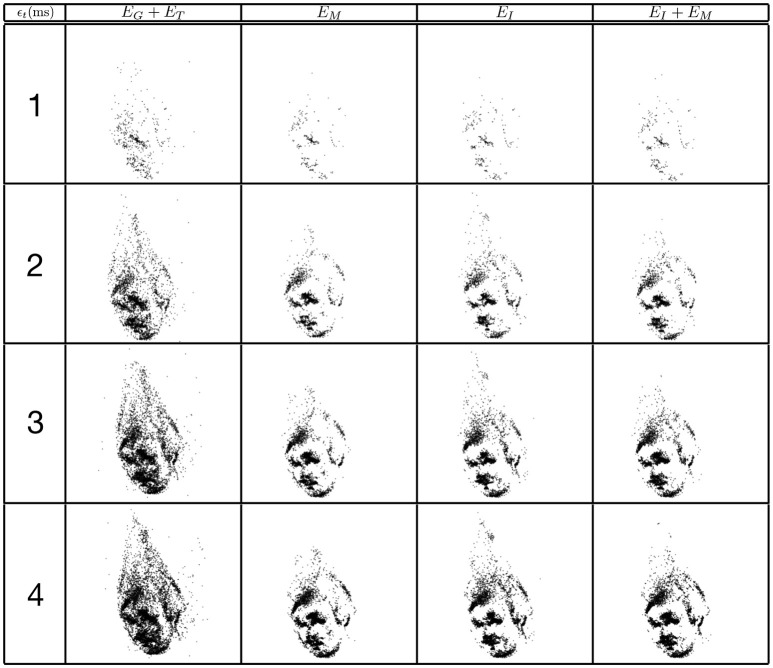
Influence of the width of the matching time window on the reconstruction accuracy using the trinocular configuration. Figures show 50ms of 3D matched events.

**Figure 9:**
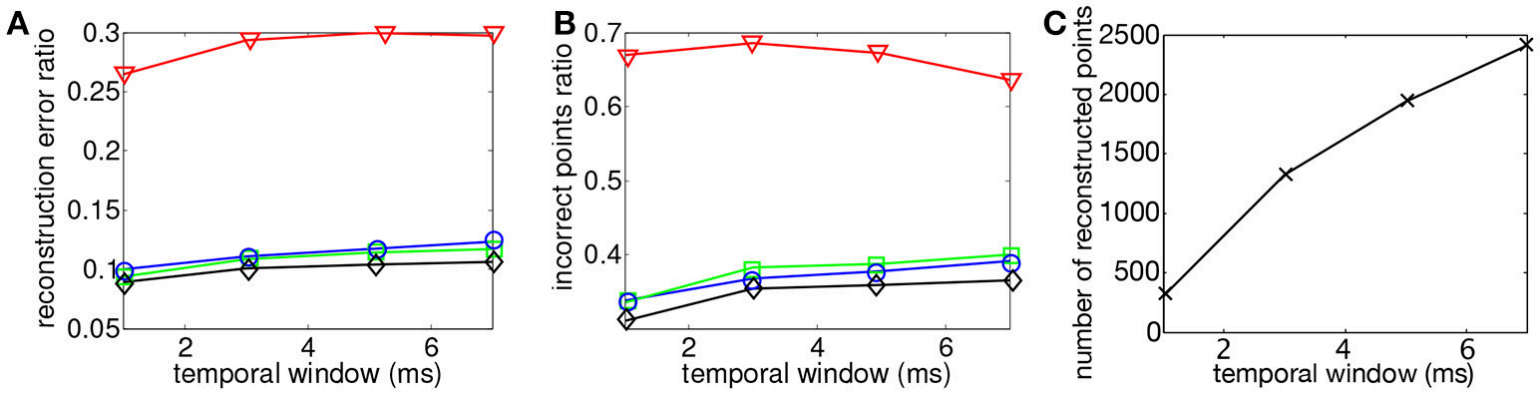
Comparison of reconstruction accuracy and errors for each constraint and variable matching time window size for a trinocular configuration. Red line (triangle) represents (*E*_*G*_ + *E*_*T*_), green line (square) the addition of motion (*E*_*M*_), blue line (circle) addition of (*E*_*I*_) and black (diamond) the addition of motion and luminance (*E*_*M*_ + *E*_*I*_). **(A)** Accuracy of 3D reconstruction **(B)** Amount of incorrect points **(C)** Total amount of reconstructed points.

The same evaluation is performed for the four criterions. Figure 8 shows the reconstruction of a face with matching time-windows ϵ_*t*_ ranging as follows 1 ms ≤ ϵ_*t*_ ≤ 7 ms at fixed intervals of 2 ms and fixed a ϵ_*g*_ = 1 pixel. Quantitative evaluation is summarized in Figure 9.

Comparing to the results obtained from the binocular configuration, we can see that the accuracy of 3D reconstructions almost doubles with the trinocular method, with reconstruction errors decreasing from 50% to 25–30% for *E*_*G*_ + *E*_*T*_ and decreasing from 20–25% to 10% for the remaining cost functions. The amount of noise also decreases in the trinocular case with 65% of wrong matches for the basic criterion *E*_*G*_ + *E*_*T*_ and around 35–40% when additional constraints are added. An improvement obtained with the trinocular formulation is an expected result as using three cameras introduces more constraints. Due to the non idealities of the sensors, the appearance of events is stochastic, the amount of reconstructed points is then lower as the method requires three corresponding events to be output inside the matching time window.

**Figure 10:**
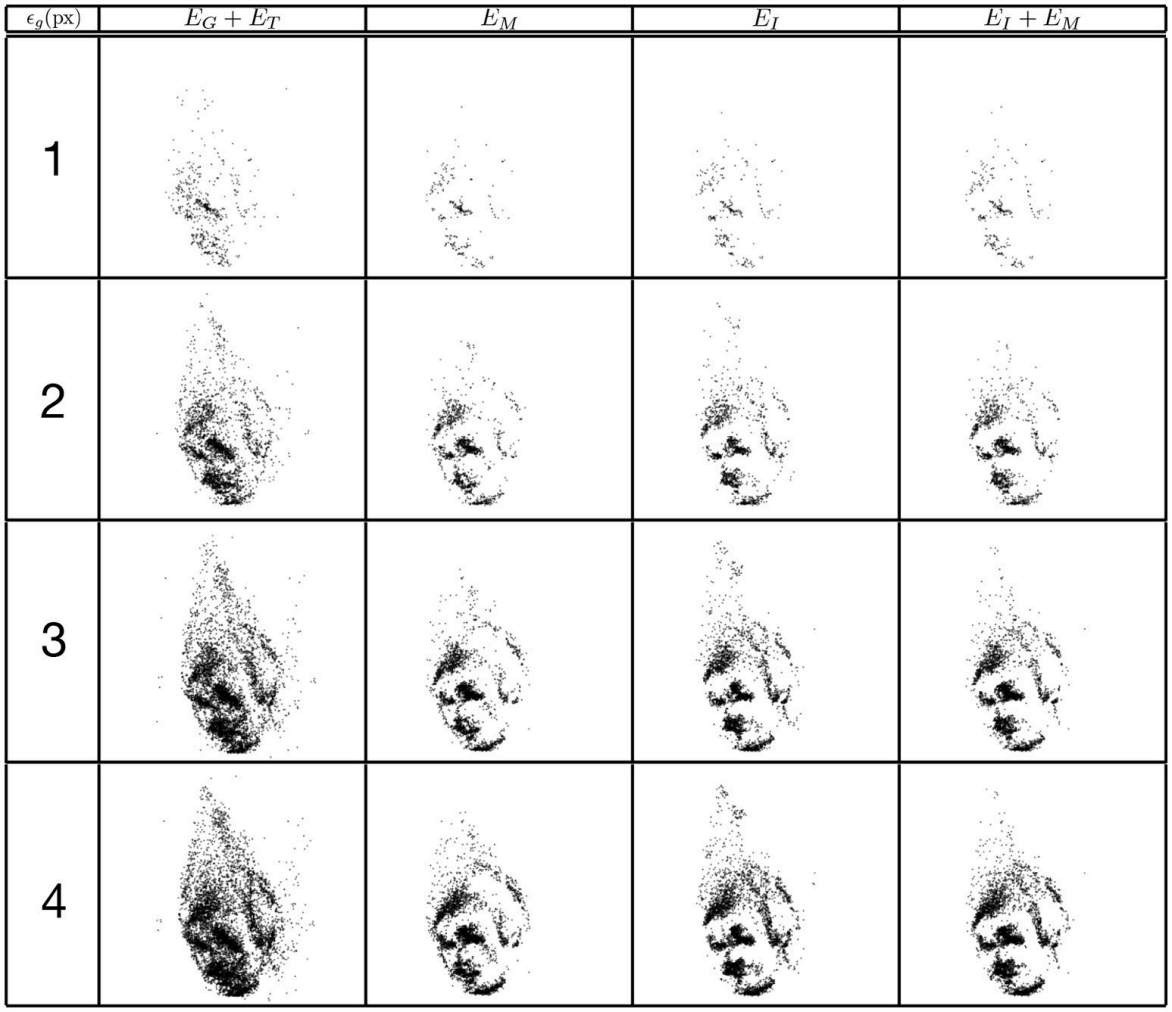
Influence of matching pixel error on reconstruction results using the trinocular event-based method. The reconstruction is achieved with a time window of 1ms. Figures are created from 50ms of 3D events.

**Figure 11:**
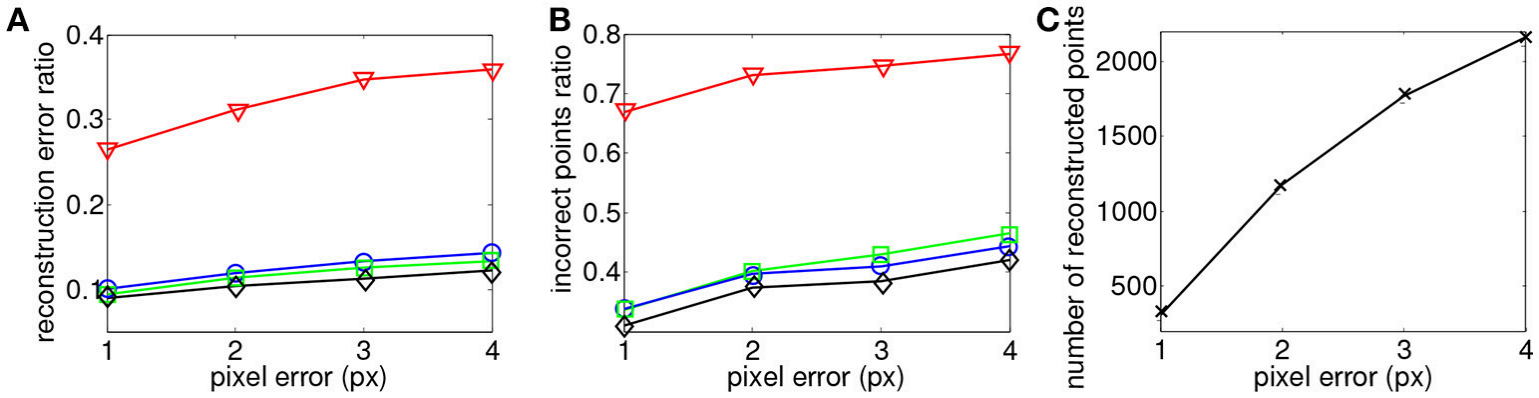
Comparison of reconstruction accuracy with each constraint and variable geometrical distances with asynchronous event-based trinocular stereo matching. Red line (triangle) represents geometrical and temporal minimization (*E*_*G*_ + *E*_*T*_), green line (square) represents motion minimization (*E*_*M*_), blue line (circle) represents luminance minimization (*E*_*L*_) and black (diamond) represents motion and luminance minimization (*E*_*M*_ + *E*_*L*_). **(A)** Accuracy of 3D reconstruction **(B)** Amount of incorrect points **(C)** Total amount of reconstructed points.

Finally, Figure 10 presents reconstructions using the asynchronous event-based trinocular stereo matching algorithm for several geometrical distance 1 pixel ≤ ϵ_*g*_ ≤ 4 pixel and for a fixed time window ϵ_*t*_ = 1 ms. A large value of ϵ_*g*_ produces noisier reconstructions particularly noticeable in *E*_*G*_ + *E*_*T*_. Figure 11 shows the comparison between the computed 3D clouds and the ground truth generated by the Kinect. There is an increase of the accuracy of the reconstructions similarly to what has been shown when studying the possibility of the matching time window. However it is interesting to notice that the reconstruction error and noise seem to increase when the acceptable distance to the epipolar lines is increased suggesting that the trinocular algorithm is more sensitive to spatial constraints. Finally, it must be highlighted that results from binocular and trinocular experiments show that the use of any of the proposed additional criterion at least doubles the accuracy of reconstructions when compared to the basic criterion used alone. Furthermore the noise of reconstructions introduced by wrong matches is also reduced by 20 to 50% implying more accurate reconstructions.

### 4.3. Computing times

**Figure 12:**
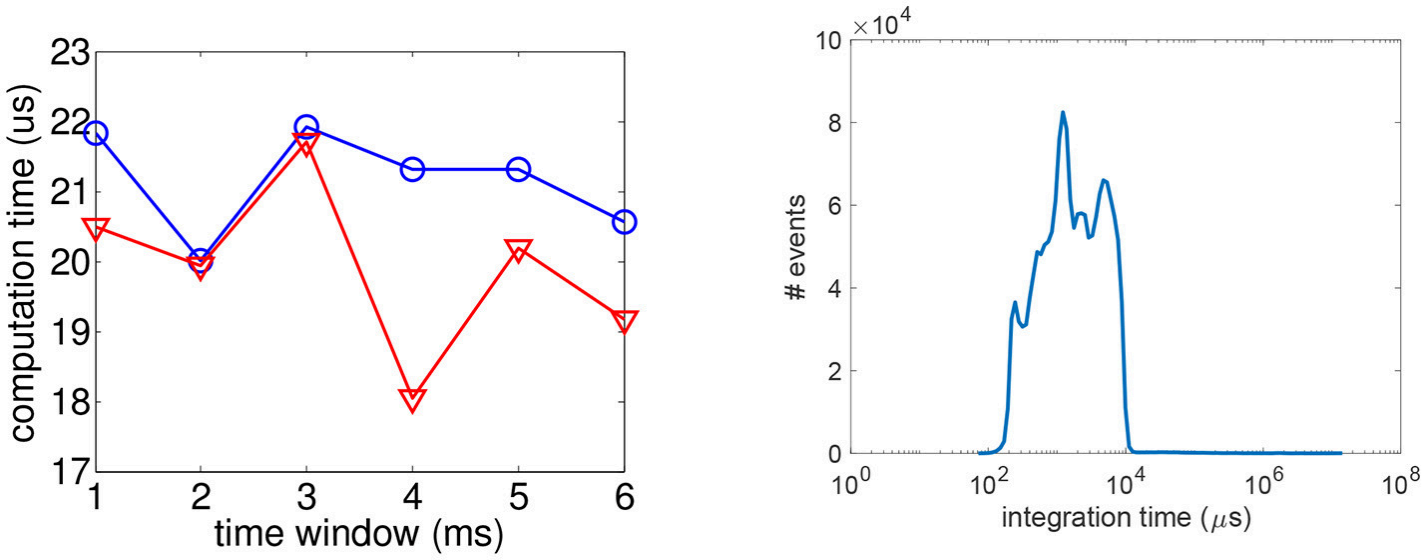
**(Left)** Average time to compute a pair (red) or a 3-tuple (blue) w.r.t. the time windows length. **(Right)** Distribution of the pixels' integration time for the tested sequence.

To evaluate the computing times to mach events, we have to define first the resources used to implement the algorithms. All the codes have been implemented in C++, on an 8GB computer powered by an I7-2630QM CPU. Then, from the data perspective, the computation time is mainly influenced by the temporal windows of search since the increase of its size is introducing more candidate to test in the minimization process. We measured for the time windows length increasing from 1 to 6 ms, the average time to match a pair of event for a binocular system and for a trinocular system. The mean computing time is calculated by averaging over the four matching criterions. Going beyond a time windows of 6 ms is not interesting as the reconstruction error is becoming to high. As shown by Figure 12 (left), for that range of temporal windows the computation time is rather stable: around 20 μs for the binocular system and 21 μs for the trinocular one. This is equivalent to be able to match 47–49 k pairs or 3-tuples per second. This is to be compared to the Kinect we used to provide the ground truth: this depth sensor when working at the resolution of 320 × 240 pixels, is providing dense depth maps at a frequency of 30 Hz, hence it is able to process up to 320 × 240 × 30 ≈ 2.3M pixel/sec. In that respect, the Kinect is performing better than the event-based alternative.

The event-based sensor does not impose a global exposure time to the pixels, however each individual pixel has an luminance integration time which is mainly scene dependent. This is adding to the processing chain a latency. We have established the integration time distribution as shown in Figure 12 (right): the peak is reached for 1,200 μs , for a mean integration time of 4,000 μs. In the log scale representation, one can see that the significative intgeration time varies from 100 to 10,000 μs. All these integration times have been measured in a standard office lighting condition (around 1,000 lux). This data acquisition latency is significantly small compared to a 100 fps camera that acquired a new frame every 10ms. For the event-based vision sensors, the latency of 10ms is reached only marginaly by some pixels at some given time in the tested sequence. As the latency due to the acquisition is larger than the computing time, the presented algorithm is processing the stream of events in realtime.

## 5. Discussion

The introduction of neuromorphic silicon retinas, bio-inspired vision sensors which encode visual information as a stream of events provides a new way to address the stereo correspondence problem. Early solutions such as Rogister et al. (2011) and Carneiro et al. (2013) used classical epipolar geometry and the precise timing of these sensors to match events and recover depth in an asynchronous event-based fashion. However, these methods were prone to errors as ambiguities could not be solved from co-activation and geometry alone.

We studied temporal-based constraints with luminance and motion information expressed in terms of time. We proposed independent energy cost functions for each of the four constraints: geometry, time, motion and luminance. We introduced a modular formulation of an energy cost function composed by any combination of the available matching cost functions. This modular approach has the advantage of allowing to chose energy cost functions according to available information or performance concerns. Furthermore proposed constraints (luminance and motion) were defined as functions of time allowing the asynchronous event-based stereo correspondence problem to be described as the minimization of an energy cost function solely dependent on the variable time. It is interesting to notice that the motion criterion is somehow embedding the temporal information, and as we can see, the use of the time surface improve significantly the matching performances. If the motion is used, it might be possible to save memory and computation ressources by skipping the *E*_*T*_ quantity and just focuse on the other constraints.

We show that the added luminance constancy and motion consistency cost functions greatly increase accuracy of reconstructions while reducing the amount of wrong matches and noise in both binocular and trinocular versions. Results prove that complex shapes can be reconstructed with high accuracy when luminance or motion minimization are used.

The presented event-based matching algorithm requires constant balance between accuracy and the time one can allocated to the computation to reach the best accuracy possible: relaxing error tolerances increases the number of reconstructed points and obviously the errors. Temporal windows length is hard to set as it is scene dependent, it can be infered from the motion information that first need to be extracted from the data. This is unavoidably requiring processing power, however some acceptable length can be chosen based on statistical observation of similar scenes. The spatial resolution is also an important limitation, the DVS and the ATIS have poor spatial resolutions compared to modern vision sensors. Typical tolerated erorrs are from 1 to 2 pixels in practice, increasing this just slow down the processing time and increase reconstruction error. The relaxation of these errors should be acceptable only when there is not enough events for point reconstruction.

The experiments have been performed with the ATIS because of its ability to deliver time encoded gray levels asynchronously, however the presented method is not restricted to the ATIS as long as the constrast change events are available. This is the case for all the existing event-based vision sensors as the ones listed summarized in Delbruck (2016) since they are derived to some extend from the DVS. This list includes sensors that combine the event-based sampling with a traditional synchronous frame acquisition mechanism (Berner et al., 2013; Brandli et al., 2014) to acquire also gray levels. The generalized time-based technique does apply to the events output by these sensors but as it is, it cannot yet integrate the synchronous luminance information.

## Author contributions

S-HI: drafting the work and revising it critically for important intellectual content, agree to be accountable for all aspects of the work in ensuring that questions related to the accuracy or integrity of any part of the work are appropriately investigated and resolved; JC: drafting the work, conception or design of the work, acquisition, analysis and interpretation of data for the work; MO: acquisition, analysis and interpretation of data for the work; RB: drafting the work revising it critically for important intellectual content.

### Conflict of interest statement

The reviewer YS declared a shared affiliation, though no other collaboration, with one of the authors MO to the handling Editor. The remaining authors declare that the research was conducted in the absence of any commercial or financial relationships that could be construed as a potential conflict of interest.
